# Age-Dependent Oxidative Stress Elevates Arginase 1 and Uncoupled Nitric Oxide Synthesis in Skeletal Muscle of Aged Mice

**DOI:** 10.1155/2019/1704650

**Published:** 2019-05-08

**Authors:** Chirayu D. Pandya, Byung Lee, Haroldo A. Toque, Bharati Mendhe, Robert T. Bragg, Bhaumik Pandya, Reem T. Atawia, Carlos Isales, Mark Hamrick, R. William Caldwell, Sadanand Fulzele

**Affiliations:** ^1^Department of Orthopaedic Surgery, Augusta University, Augusta, GA 30912, USA; ^2^Department of Neurosurgery, University of Kentucky, Lexington, KY 40506, USA; ^3^Department of Pharmacology and Toxicology, Augusta University, Augusta, GA 30912, USA; ^4^Department of Cell Biology and Anatomy, Augusta University, Augusta, GA 30912, USA; ^5^Georgia Cancer Center, Augusta University, Augusta, GA 30912, USA; ^6^Department of Endocrinology, Augusta University, Augusta, GA 30912, USA; ^7^Center for Healthy Aging, Augusta University, Augusta, GA 30912, USA

## Abstract

Aging is associated with reduced muscle mass (sarcopenia) and poor bone quality (osteoporosis), which together increase the incidence of falls and bone fractures. It is widely appreciated that aging triggers systemic oxidative stress, which can impair myoblast cell survival and differentiation. We previously reported that arginase plays an important role in oxidative stress-dependent bone loss. We hypothesized that arginase activity is dysregulated with aging in muscles and may be involved in muscle pathophysiology. To investigate this, we analyzed arginase activity and its expression in skeletal muscles of young and aged mice. We found that arginase activity and arginase 1 expression were significantly elevated in aged muscles. We also demonstrated that SOD2, GPx1, and NOX2 increased with age in skeletal muscle. Most importantly, we also demonstrated elevated levels of peroxynitrite formation and uncoupling of eNOS in aged muscles. Our in vitro studies using C2C12 myoblasts showed that the oxidative stress treatment increased arginase activity, decreased cell survival, and increased apoptotic markers. These effects were reversed by treatment with an arginase inhibitor, 2(S)-amino-6-boronohexanoic acid (ABH). Our study provides strong evidence that L-arginine metabolism is altered in aged muscle and that arginase inhibition could be used as a novel therapeutic target for age-related muscle complications.

## 1. Introduction

Aging is associated with reduced muscle mass (sarcopenia) and strength (dynapenia), which can increase the incidence of falls and bone fractures [1]. As the number of older adults continues to increase, the problem of muscle loss becomes a significant public health concern [1–3]. Falls and fractures in turn lead to prolonged disability, poor quality of life, and significant financial burden [4]. It is widely appreciated that aging triggers systemic oxidative stress, which can impair myoblast differentiation and cell survival, which also leads to muscle loss [5, 6]. Recent studies have shown that elevated levels of reactive oxygen species (ROS) have deleterious effects on the musculoskeletal system and are critical in muscle-related pathophysiology [5–10]. At the molecular level, generation of ROS elicits a wide range of effects on cells such as autophagy, cell differentiation and proliferation arrest, DNA damage, and cell death by activation of numerous cell signaling pathways [5, 6]. We previously reported that oxidative stress decreases cell attachment, proliferation, and migration of bone marrow stromal cells and antioxidant supplementation can reverse these effects [11].

We recently reported that bone marrow stromal cells express arginase 1 (ARG1) and its expression is regulated by high glucose [12]. Arginase is an enzyme which metabolizes L-arginine to form urea and L-ornithine in the urea cycle. There are two known arginase isoforms: arginase 1 (ARG1) and arginase 2 (ARG2). ARG1 is a cytosolic enzyme and is expressed most abundantly in the liver where it plays a vital role in the urea cycle, while ARG2 is located in mitochondria of various cell types [13]. Arginine is a semiessential amino acid which is the substrate for both nitric oxide synthase (NOS) and arginase enzyme. ARG1 is known to regulate oxidative stress in various degenerative diseases by modulating nitric oxides (NO) [14–16]. Recent studies indicated that NO is one of the important therapeutic targets for a number of cardiovascular and age-related diseases [17–19]. Our laboratory previously reported that ARG1 expression is elevated in diabetic bone and bone marrow [12]. Furthermore, diabetic bones were osteoporotic in nature. Interestingly, we found that treatment with the ARG1 inhibitor improved the quality of diabetic mice [12]. Based on our previous studies, we speculate that ARG1 becomes dysregulated in aged muscle. Until now, little is known about the role of oxidative stress in ARG1 regulation in aging muscle.

In the present study, we investigated the arginase activity and arginase 1 expression in aged muscles. We also analyzed the expression of important oxidative stress-related signaling molecules in muscles of aged mice. Furthermore, we performed in vitro studies on the myoblast cell line (C2C12) and arginase inhibitor (ABH) to investigate the role of arginase in myoblast pathophysiology. We found elevated levels of ROS accumulation and ARG1 expression/arginase activity and uncoupling of eNOS in aged muscles. Additionally, our in vitro studies showed that the arginase inhibitor prevented the formation of ROS accumulation and NOS uncoupling in myoblasts and improves the physiological health of myoblast cells.

## 2. Material and Methods

### 2.1. Animal Preparation and Experimental Design

All animal protocols were approved by the Institutional Animal Care and Use Committee at Augusta University. Male C57BL/6 mice from 3 months and 22 months of age (10 mice per age group) were obtained from the aged rodent colony at the National Institute on Aging. Animals were housed in a 12 h light/dark cycle and had free access to food and water throughout the study. Mice were sacrificed, and the quadriceps muscle was dissected free from the hindlimb and used for protein isolation for arginase activity, western blot, and RNA isolation.

### 2.2. Arginase Activity Assay

Muscle homogenate lysates were prepared in Tris buffer (50 mM Tris HCl, 0.1 mmol/L EDTA and EGTA (pH 7.5), containing protease inhibitors) and were used for the arginase activity assay as previously described [12]. Briefly, 25 *μ*L of 10 mM MnCl2 was added to 25 *μ*L of homogenates (cell or tissue) and heated at 57°C for 10 min to activate arginase. Next, 50 *μ*L of 0.5 M L-arginine was then added to the reaction tube and incubated at 37°C for 1 h, and 400 *μ*L of acid mixture (H_2_SO : H_3_PO4 : H_2_O in a ratio of 1 : 3 : 7) was added to stop the reaction. Then, 25 *μ*L of 9% a-isonitrosopropiophenone (in ethanol) was added, and the mixture was heated for 45 min at 100°C and placed in the dark for 10 min to develop color. Arginase activity was measured by loading 200 *μ*L of the reaction mixture in a 96-well plate, and absorbance was read at 540 nm.

### 2.3. Isolation of RNA, Synthesis of cDNA, and Real-Time PCR

Real-time PCR was performed as per our published method [11, 12]. Total RNA was isolated from the quadriceps muscles of mice. The muscle was homogenized and dissolved in TRIzol. RNA was isolated using the TRIzol method following the manufacturer's instructions, and the quality of the RNA preparations was monitored by absorbance at 260 and 280 nm (Helios Gamma, Thermo Spectronic, Rochester, NY). The RNA was reverse-transcribed into complementary deoxyribonucleic acid (cDNA) using iScript reagents from Bio-Rad on a programmable thermal cycler (PCR Sprint, Thermo Electron, Milford, MA). The cDNA (50 ng) was amplified by real-time PCR using a Bio-Rad iCycler and ABgene reagents (Fisher Scientific, Pittsburgh, PA) and ARG1 primers [12]. Glyceraldehyde-3-phosphate dehydrogenase (GAPDH) was used as the internal control for normalization.

### 2.4. Western Blot Analysis

Protein was extracted from quadriceps muscle and cell culture lysate, subjected to SDS-PAGE, and transferred to nitrocellulose membranes. Membranes were incubated with a polyclonal antibody against glutathione peroxidase (GPx1), superoxide dismutase (SOD2) (Santa Cruz Biotechnology, Santa Cruz, CA), NOX2, 3-NT, eNOS (Santa Cruz Biotechnology, Santa Cruz, CA), and GAPDH (Santa Cruz Biotechnology, Santa Cruz, CA) overnight at 4°C, followed by incubation with an appropriate secondary antibody. Proteins were visualized with an ECL western blot detection system (Thermo Scientific, Waltham, MA). For detection of eNOS dimers, we ran a low-temperature SDS-PAGE (LT-PAGE) gel using reported procedures [20] with slight modification. The protein lysates were prepared using 1× Laemmli buffer without 2-mercaptoethanol. The samples were then subjected to SDS-PAGE with 7.5% gel and run at a low temperature by keeping the buffer tank surrounded by ice. The gels were transferred, and the blots were probed as described above.

### 2.5. Arginase Inhibitor Prevents C2C12 Cells from Oxidative Stress Damage

C2C12 cells were cultured and pretreated with or without the arginase inhibitor ABH (100 *μ*M) for 4 h followed by hydrogen peroxide (100 *μ*M) treatment alone or in combination with ABH for 24 h. Arginase activity, RT-PCR, MTT assay, and staining were performed as described below. Superoxide and hydrogen peroxide levels were detected in culture cells with dihydroethidium (DHE) staining dye as previously described [21, 22]. C2C12 cells were incubated with the DHE dye mentioned above in PBS for 30 min at 37°C. Fluorescence was monitored using a fluorescence microscope at 20x magnification.

### 2.6. Cell Survival Assay

To investigate the effect of oxidative stress on C2C12 cell survival, the CellTiter 96® AQueous One MTS Cell Assay kit (Promega, G3580) was used as per the published method [23, 24]. After cell culture treatment (as described above), cells were washed twice with PBS and 150 *μ*L of MTS (CellTiter 96® AQueous One Solution Reagent, Promega) assay buffer was added. Cells were then incubated for 2 h at 37°C in a humidified 5% CO_2_ incubator. Optical density (OD) was read at 490 nm.

### 2.7. Statistical Analysis

GraphPad Prism 5 (La Jolla, CA) was utilized to perform one-way ANOVA with Bonferroni pairwise comparison or unpaired *t*-tests as appropriate. A *p* value of <0.05 was considered significant.

## 3. Results

### 3.1. Elevated Level of Arginase Activity and Expression in Aged Muscles

**Figure 1:**
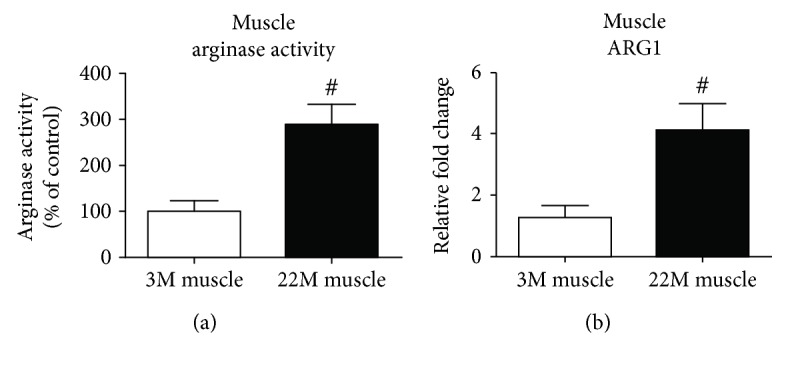
Aging increases arginase activity and mRNA expression in muscles. (a) Arginase activity was determined using an assay for urea formation in muscle lysates from young and old muscles, and (b) real-time PCR analysis of Arg1 mRNA in young and old mice. Data for each sample were normalized to GAPDH mRNA and represented as the fold change in expression compared to young mice. Results are means ± SD (*n* = 5-6 ^#^*p* < 0.01); data were analyzed using an unpaired *t*-test.

Our published data demonstrated that chronic oxidative stress increased arginase activity in various disease conditions [12, 21, 22]. We hypothesized that arginase expression and activity became dysregulated in aged muscles. Our data showed that this is indeed the case that arginase activity (*p* value = 0.01) and arginase 1 expression (*p* value = 0.01) were significantly elevated in 22-month-old aged muscle (Figure 1). Previously, our group reported that muscle mass declined significantly between 18 and 24 months of age [25].

### 3.2. Elevated Level of Oxidative Stress in Aged Muscle

To evaluate the activities of the antioxidant defense system in aged muscles, we determined the level of superoxide dismutase 2 (SOD2) and glutathione peroxidase 1 (GPx1). SOD2 and GPx1 are antioxidant enzymes that play a vital role in the suppression or prevention of the formation of free radical or reactive species in cells and tissues [26, 27]. Our western blot data showed that SOD2 (*p* value = 0.05) and GPx1 (*p* value = 0.001) antioxidant enzymes significantly (*p* value = 0.04) increased in the muscle from 22-month-old mice compared to 3-month-old young animals [28, 29].

**Figure 2:**
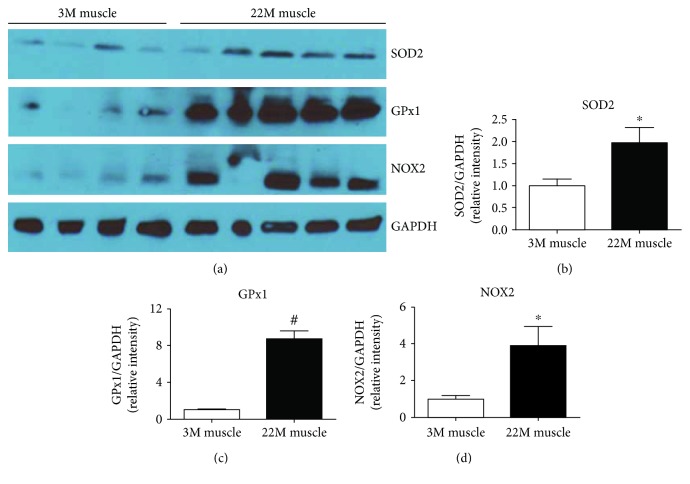
Elevated level of oxidative stress in young and old mouse muscles. (a) Representative western blots of protein extracted from young and old muscle samples. Densitometry quantification of (b) SOD2, (c) NOX2, and (d) GPx1. Values are normalized to the expression levels of the housekeeping gene GAPDH. Results are means ± SD (*n* = 5-6, ^∗^*p* < 0.05, ^#^*p* < 0.01); data were analyzed using an unpaired *t*-test.

NADPH oxidase is one of the important enzymes known for generation of reactive oxygen species with age [28, 29]. We analyzed NOX2 (gp91-phox) levels in young (3 months) and old (22 months) mouse muscle samples. We found a significant (*p* value = 0.039) increase in NOX2 level in old muscles compared to young muscles (Figure 2).

### 3.3. Increased Peroxynitrite and ROS in Aging Muscles

**Figure 3:**
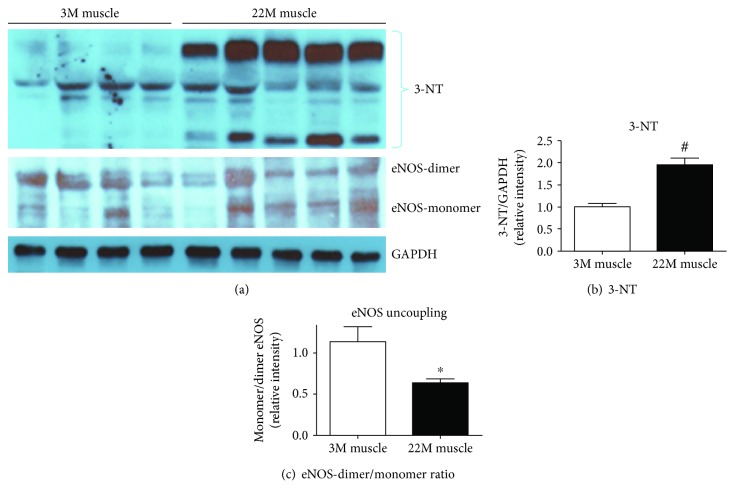
Elevated level of peroxynitrite (ONOO^−^) formation and uncoupling of eNOS in aged muscle. (a) Representative western blots of protein extracted from young and old muscle samples for 3-NT and eNOS uncoupling. Densitometry quantification of (b) 3-NT and (c) eNOS uncoupling. Values are normalized to the expression levels of the housekeeping gene GAPDH. Results are means ± SD (*n* = 5-6, ^∗^*p* < 0.05, ^#^*p* < 0.01); data were analyzed using an unpaired *t*-test.

Aging affects the ROS and reactive nitrogen species (RNS) homeostasis, which leads to musculoskeletal-related complications. ROS and RNS play important roles in various age-related diseases including sarcopenia [5, 6, 8–10]. We investigated the RNS status in aged muscle using 3-nitrotyrosine (3-NT), a specific marker for reactive nitrogen species [30]. Our data demonstrated that 22-month-old muscles have significantly (*p* value = 0.0041) higher levels of 3-NT compared to young muscles (Figures 3(a) and 3(b)). Elevated levels of 3-NT in aged muscles suggest activation of the nitrating pathway and production of increased reactive nitrogen intermediate products.

### 3.4. Dysregulation of the Monomer-to-Dimer Ratio (Uncoupling) of eNOS in Aged Mouse Muscles

Previously, eNOS uncoupling is related to several age-related diseases [20, 31–33]. We hypothesized that eNOS might be uncoupled because of the elevated level of oxidative stress in aged muscles. We analyzed the eNOS monomer and dimer in young and old muscles using a low-temperature SDF-PAGE gel. We found significant (*p* value = 0.04) uncoupling of eNOS in aged muscles (Figures 3(a) and 3(c)). The ratio of eNOS monomer to dimer was significantly higher in aged muscles compared to young muscles (Figures 3(a) and 3(c)).

### 3.5. Oxidative Stress Regulates Arginase Activity in Myoblasts (C2C12 Cells)

**Figure 4:**
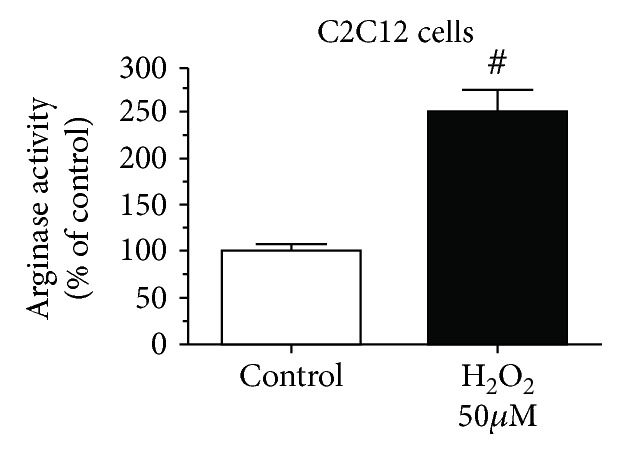
Effect of oxidative stress on arginase activity on myoblasts. C2C12 cells were incubated in DMEM (2% FBS, 50 mM L-arginine) with and without hydrogen peroxide (50 *μ*M) for 48 h. Arginase activity in cell lysate was determined by the arginase activity assay (^#^*p* < 0.01, *n* = 6).

**Figure 5:**
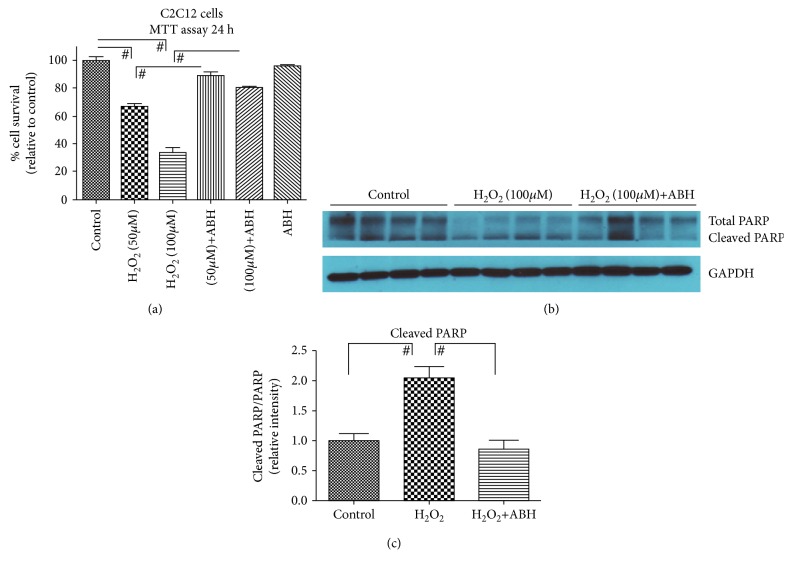
Arginase inhibitor prevents C2C12 cells from oxidative stress damage. (a) C2C12 cells were treated with H_2_O_2_ (50 and 100 *μ*M) in the presence or absence of ABH (100 *μ*M) for 24 h. MTS analysis was performed after 24 h following treatment. (b) Representative western blots for total PARP and cleaved PARP on C2C12 cells. (c) Densitometry ratio of total PARP and cleaved PARP. Values are normalized to the expression levels of the housekeeping gene GAPDH. Data were analyzed by one-way ANOVA followed by the Bonferroni post hoc test (^#^*p* < 0.01, *n* = 8).

Our data (Figure 1) demonstrates that arginase activity and ARG1 expression are upregulated in muscles with aging. To further demonstrate the role of arginase in oxidative stress-dependent myoblast biology and cell survival, we treated C2C12 cells with hydrogen peroxide (oxidative stress) and estimated arginase activity. We found significantly (*p* value = 0.01) elevated levels of arginase activity in H_2_O_2_-treated cells (Figure 4). Based on this data, we hypothesized that an arginase inhibitor might prevent cells from the harmful effects of oxidative stress. We subjected cells to oxidative stress in the presence or absence of the arginase inhibitor (ABH), performed a cell survival assay, and investigated cell apoptotic markers. The cell survival assay was performed using the MTS assay, in which we found a dose-dependent significant (*p* value = 0.001) decrease in the C2C12 cell number in oxidative stress samples, and the arginase inhibitor prevents cell death (*p* value = 0.001) (Figure 5(a)). Western blot analysis of the apoptotic marker (cleaved PARP) showed a significant (*p* value = 0.01) increase in cleaved PARP in the presence of oxidative stress. Treatment with the arginase inhibitor prevented this effect (Figures 5(b) and 5(c)).

### 3.6. Arginase Inhibitor Prevents Superoxide Radical Formation in C2C12 Cells

**Figure 6:**
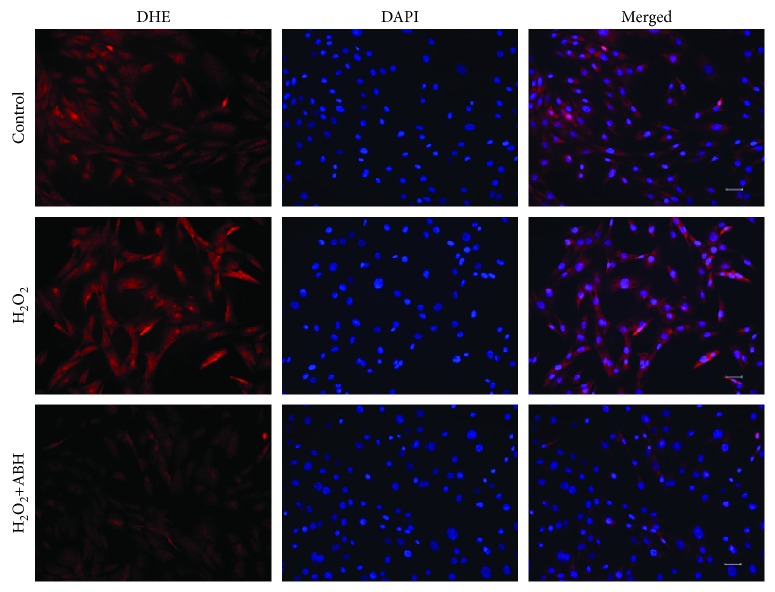
Fluorescence microscopy images show that the arginase inhibitor prevents the accumulation of ROS in C2C12 cells. C2C12 cells were treated with H_2_O_2_ (50 *μ*M) in the presence or absence of ABH (100 *μ*M) for 24 h. ROS production was detected by DHE staining. Representative fluorescent images show that the arginase inhibitor prevents the accumulation of ROS in C2C12 cells.

To assess the involvement of arginase in myoblasts during oxidative stress, we performed *in vitro* studies using C2C12 cells. C2C12 cells were cultured and treated with H_2_O_2_ to induce oxidative stress in the presence or absence of the arginase inhibitor (ABH) and stained with DHE staining dye. Dihydroethidium (DHE) is a cell-permeable dye that reacts with a superoxide anion and forms a red fluorescent product [34]. Our data showed increased red fluorescence in oxidative stress-subjected samples. Increased fluorescence of DHE staining in the cells revealed increased superoxide radical formation. Furthermore, treatment with ABH (Figure 6) prevented the increase in DHE fluorescence indicating the prevention of superoxide production in cells.

## 4. Discussion

Aging affects the homeostasis of ROS, which is characterized by the increased accumulation of intracellular hydrogen peroxide/RNS and decreased antioxidant properties of cells/tissues. Imbalance (generation and elimination) in the homeostasis of ROS leads to musculoskeletal pathophysiology, such as muscle atrophy and fibrosis [6, 35]. When ROS levels are above the physiologic level, cells respond to stress through compensatory mechanisms by increasing antioxidant signaling to prevent the damaging effects of ROS. In chronic stress conditions, such as aging, ROS levels continue to increase while antioxidant systems become hampered, leading to muscle atrophy and fibrosis. Previously, our group and others have shown accumulation of reactive oxygen species and muscle loss with age [6, 25, 35–38]. We hypothesized that with aging, elevated levels of oxidative stress increase arginase activity and ARG1 expression in muscle.

In this study, we analyzed the expression of arginase 1 and its activity in young and aged muscles. Our study is the first to demonstrate both arginase activity and expression elevation with age in muscles. Recently, we demonstrated the elevated levels of oxidative stress and ARG1 expression in diabetic mouse bone and bone marrow [12]. Furthermore, our group also demonstrated ARG1 dysregulation in various tissues and organs of diabetic and hypertension disease models [12, 21, 22, 39–42].

Previously, our group reported that NADPH oxidase 2 (NOX2) activation led to elevated ROS and ARG1 expression and activity in diabetic retinal endothelial dysfunction [43]. NADPH oxidase plays an important role in the production of superoxide free radicals to protect cells from foreign microorganisms [44]. Controlled regulation of NADPH oxidase is important to maintain the health level of ROS. Chronic stress continuously elevates NADPH oxidase, which is harmful for cells and induces degenerative effect [43]. We speculate that NADPH oxidase 2 expression might be affected by aging. To investigate this, we assessed the expression of NADPH oxidase 2 (NOX2). As expected, we found elevated level of NOX2 expressed in aged muscles compared to young muscles. Whitehead and his group [45] previously reported elevated NADPH oxidase expression in tibialis anterior muscles from dystrophic (mdx) mice. Similar results were also reported by Heymes et al. [46] in the pathophysiology of human congestive heart failure. We hypothesized that aging-induced oxidative stress (e.g., NOX2) elevates arginase expression, thus limiting L-arginine bioavailability and reducing NO production in muscle. Elevated arginase activity can limit the bioavailability of L-arginine to NOS causing its uncoupling, which results in less NO formation and more superoxide (O2^·-^) production. The NO rapidly reacts with O2^·-^ to produce peroxynitrite (ONOO^−^), another potent oxidant [47]. It has been well established that peroxynitrite participates in oxidation reactions, which results in the modification of amino acid residue of proteins (protein tyrosine nitration) leading to degenerative changes [48, 49]. We speculate that in aged muscles, peroxynitrite level might be higher than that in young muscles. We analyzed the presence of 3-nitrotyrosine (3-NT) in young and old muscles, which indirectly measures the presence of peroxynitrite [50]. We found increased level of 3-NT in aged muscle compared to young muscle. Pearson et al. [51] also reported similar findings with ours showing elevated level of 3-NT in gastrocnemius muscles of old mice.

Formation of peroxynitrite and superoxide affects eNOS uncoupling in various age-related diseases [20, 31–33]. In this study, we demonstrated that the eNOS monomer-to-dimer ratio is disturbed in aged muscles. To the best of our knowledge, ours is the first study to directly demonstrate eNOS uncoupling in aged muscle. Low-temperature SDF-PAGE gel demonstrated a higher eNOS monomer-to-dimer ratio in aged muscles. For the effective function of eNOS, dimerization of eNOS is required, which catalyzes the L-arginine to generate NO [52]. Our *in vitro* data further confirm the above findings; we used mouse myoblast (C2C12) cell lines to perform these studies. Subjecting C2C12 cells to oxidative stress resulted in elevated level of ROS and arginase activity, and pretreatment with the arginase inhibitor reversed the effects suggesting the role of arginase in myoblast pathophysiology. We also found that oxidative stress decreases C2C12 cell survival and increases cell apoptosis, while the arginase inhibitor prevented this effect.

Overall, our study showed that aging elevates arginase activity, which contributes to less NO production due to competition for L-arginine and eNOS uncoupling. Further studies are needed to fully understand the mechanism of age-induced increases in arginase activity and its specific role in uncoupling of eNOS. Our study demonstrated that limiting arginase activity in muscle with aging can prevent or slow down the degenerative effect. Further studies are needed to investigate the therapeutic role of the arginase inhibitor in age-related muscle loss/complications. The aging population is at increased risk of falls and fractures due to low muscle mass and strength [1–3]. As the number of older adults continues to increase, the problem of muscle loss becomes a significant public health concern. Our study outcome has a significant translational impact because it suggested that the arginase inhibitor could be used as a novel therapeutic target for age-related muscle loss.

## Data Availability

The quantitative data used to support the findings of this study will be available from the corresponding author upon request.
